# Differential S1P Receptor Profiles on M1- and M2-Polarized Macrophages Affect Macrophage Cytokine Production and Migration

**DOI:** 10.1155/2017/7584621

**Published:** 2017-03-06

**Authors:** Jan Müller, Wolfram von Bernstorff, Claus-Dieter Heidecke, Tobias Schulze

**Affiliations:** ^1^Department of General Surgery, Visceral, Thoracic and Vascular Surgery, Universitätsmedizin Greifswald, Ferdinand-Sauerbruch-Str., 17475 Greifswald, Germany; ^2^Department of Vascular Surgery, Krankenhaus Ludmillenstift, Ludmillenstraße 4-6, 49716 Meppen, Germany

## Abstract

*Introduction*. Macrophages are key players in complex biological processes. In response to environmental signals, macrophages undergo polarization towards a proinflammatory (M1) or anti-inflammatory (M2) phenotype. Sphingosine 1-phosphate (S1P) is a bioactive lysophospholipid that acts via 5 G-protein coupled receptors (S1P_1–5_) in order to influence a broad spectrum of biological processes. This study assesses S1P receptor expression on macrophages before and after M1 and M2 polarization and performs a comparative analysis of S1P signalling in the two activational states of macrophages.* Methods*. Bone marrow derived macrophages (BMDM) from C57 BL/6 mice were cultured under either M1- or M2-polarizing conditions. S1P-receptor expression was determined by quantitative RT-PCR. Influence of S1P on macrophage activation, migration, phagocytosis, and cytokine secretion was assessed in vitro.* Results*. All 5 S1P receptor subclasses were expressed in macrophages. Culture under both M1- and M2-polarizing conditions led to significant downregulation of S1P_1_. In contrast, M1-polarized macrophages significantly downregulated S1P_4_. The expression of the remaining three S1P receptors did not change. S1P increased expression of iNOS under M2-polarizing conditions. Furthermore, S1P induced chemotaxis in M1 macrophages and changed cytokine production in M2 macrophages. Phagocytosis was not affected by S1P-signalling.* Discussion*. The expression of different specific S1P receptor profiles may provide a possibility to selectively influence M1- or M2-polarized macrophages.

## 1. Introduction

Macrophages are both key regulators and effector cells of the immune system, play prominent roles in tissue remodelling and repair, and orchestrate metabolic functions in almost all tissues of the body [1]. Macrophages in various organs show considerable heterogeneity [2]. Functionally, macrophages can be classified in classically activated, proinflammatory M1 macrophages and alternatively activated anti-inflammatory M2 macrophages [3–5]. M1 macrophages are endued with the capacity to produce high levels of proinflammatory cytokines as well as reactive oxygen and nitrogen species. They promote and amplify T_H_1 type responses, are implicated in the host defence against intracellular pathogens, and possess antitumor activity [4]. M2 macrophages are characterized by their high phagocytic capacity, the expression of scavenging molecules and decoy receptors for T_H_1-polarizing cytokines, and the efficient nitrogen metabolism through the arginase pathway leading to ornithine production required for healing and tissue repair [4, 6–8].

Macrophage differentiation and function is subjected to a tight control by cytokines and chemokines. Also, other molecules like pathogen associated-molecular patterns, danger-associated-molecular patterns, and various ligands of G-protein coupled receptors, for example, lysophospholipids, can influence macrophage differentiation and function [3, 4, 9–11].

Sphingosine-1-phosphate is a highly active lysophospholipid that plays a crucial role in the regulation of the immune response under both physiological and pathological conditions. It exerts its biological action as an extracellular messenger via G-protein coupled receptors on the cell membrane. Five different S1P binding receptors (S1P_1–5_) have been identified so far. The effects of S1P receptor-mediated signalling are particularly well described in the adaptive immune systems, where S1P gradients profoundly affect T- and B-cell trafficking and positioning within primary and secondary lymphoid organs as well as dendritic cell- (DC-) T-cell interaction [12–15]. S1P receptor expression on macrophages has been mainly determined by RT-PCR due to technical difficulties with protein detection. Expression profiles of the known five S1P receptors vary considerably depending on the organ and species origin of the cells analyzed [16–21]. However, S1P signalling affects a myriad of biological functions in various macrophage populations under physiological and disease conditions [10, 22–24].

To date, it is unknown whether S1P receptor expression and the biological action of S1P vary in M1- and M2-polarized macrophages. In the present work, we determined S1P receptor expression on differentiated nonpolarized bone marrow derived macrophages (BMDM), as well as on M1-polarized and on M2-polarized BMDM. We further investigated the biological effects of S1P signalling on these different functional states of macrophage polarization.

## 2. Materials and Methods

### 2.1. Isolation of Murine Bone Marrow Stem Cells

Murine bone marrow stem cells were isolated from femur and tibia of female C57BL/6 mice aged between 12 and 14 weeks as reported in [25]. Briefly, mice were sacrificed by cervical dislocation; femurs and tibiae were removed, opened, and flushed with 6 mL sterile PBS (1x). Bone marrow was mechanically disintegrated and pushed through a 60 *μ*m nylon net filter in order to obtain single cell suspensions. The cells were washed and resuspended in sterile RPMI 1640/5% FCS (primary cell culture medium). Cells were counted, transferred onto a 6-well flat-bottomed cell culture dish at a concentration of 10^6^ cells/mL, and incubated for 6 h at 5% CO_2_ and 37°C. After 6 h the medium was removed and differentiation was induced.

### 2.2. Differentiation of Murine Bone Marrow Cells into BMDM

Isolated bone marrow stem cells were differentiated into BMDM as reported by Zhang et al. [25]. After 6 hours of primary cell culture medium was removed and 2 mL of RPMI 1640/5% FCS containing 20 ng/mL mouse M-CSF (Miltenyi Biotec, Bergisch Gladbach, Germany) was added to each well. The cells were then incubated for 7 days at 5% CO_2_ and 37°C. The differentiation medium was renewed every 36 hours.

### 2.3. Activation of BMDM under M1/M2-Polarizing Conditions

After 7 days of differentiation, cells were washed twice with sterile PBS. For M1-activation, 2 mL RPMI 1640/5% FCS containing 1 *μ*g/mL LPS (Sigma-Aldrich Chemie GmbH, Munich, Germany) + 300 U/mL IFNy (Miltenyi Biotec, Bergisch Gladbach, Germany) were added to the wells. M2-activation was induced by the addition of medium containing 40 ng/mL IL-13 (Miltenyi Biotec, Bergisch Gladbach, Germany) + 40 ng/mL IL-4 (Miltenyi Biotec, Bergisch Gladbach, Germany) instead of LPS + IFNy. Control cells were cultured with RPMI 1640/5% FCS medium only. Cells were then incubated for 24 h under these conditions as described by Mosser and Zhang [26]. In order to demonstrate successful macrophage polarization, culture samples were stained with anti-CD206 (to assess M2-polarization) and anti-iNOS (to assess M1 polarization). Representative histograms are shown in Supplementary Figure  1 in Supplementary Material available online at https://doi.org/10.1155/2017/7584621.

### 2.4. RNA-Preparation, Reverse Transcription, and Quantitative Real-Time PCR (qPCR)

RNA was extracted from primary BMDM cell cultures using the RNeasy Mini® Kit (Qiagen, Hilden, Germany) according to the manufacturer's instructions. In order to avoid gDNA contamination, an On-Column DNase Digestion step with the RNase-Free DNase set (Qiagen, Hilden, Germany) was added to the standard protocol. Purity of RNA-samples was assessed by photometric measurement of the ratio of absorbance at 260 nm and 280 nm. For reverse transcription, the QuantiTect® Reverse Transcription Kit (Qiagen, Hilden, Germany) was used according to the manufacturer's instructions. All steps were performed in biological quintuplicate. The amount of RNA used for reverse transcription was 500 ng. Specific primers for mouse S1P_1_ (sense: 5′-AACTTTGCGAGTGAGCTGGT; antisense: 5′-CTAGAGGGCGAGGTTGAGTG), S1P_2_ (sense: 5′-ATAGACCGAGCACAGCCAAC; antisense: 5′-GAGGTGGTCTCCTGCATGTC), S1P_3_ (sense: 5′-AAGCCTAGCGGGAGAGAAAC; antisense: 5′-TCAGGGAACAATTGGGAGAG), S1P_4_ (sense: 5′-GGACTTCTCGGTCACTCAGC; antisense: 5′-GGCTTGCTGTCATGTTCTCA), S1P_5_ (sense: 5′-GGAGGGACTCTCCTGGATTC; antisense: 5′-TTCCTCTGTAGCCAGCCACT), *β*-microglobulin (sense: 5′-ATTCACCCCCACTGAGACTG; antisense: 5′-GCTATTTCTTTCTGCGTGCAT), and GAPDH (sense: 5′-GGTGCTGAGTATGTCGTGGA; antisense: 5′-CCTTCCACAATGCCAAAGTT) were designed using Primer3 software and synthetized by BIOTEZ (Berlin, Germany). In order to exclude false positive signals due to contamination with gDNA, primers for S1P_1_, S1P_2_, S1P_3_, and S1P_5_ were designed as intron-spanning primers. Due to its genomic structure, primers for S1P_4_ were both situated within one single exon. Real-time PCR was performed using an Applied Biosystems 7500 real-time PCR device. A dissociation curve was run after each PCR. All samples were run in triplicate. Relative quantification was performed using the 2^−ΔΔC_T_^ method as described by [27].

### 2.5. Flow Cytometric Staining of BMDM Activation Markers

Nonspecific binding of the specific antibody was blocked with an anti-Fc*γ*RIII/Fc*γ*RII antibody (anti-CD16/32; BD Bioscience, Heidelberg, Germany) diluted 1 : 100 in PBS at room temperature for 15 min. If an unlabelled primary Ab was used, washing steps were followed by incubation with an appropriate secondary antibody. The following FITC-, PerCp-Cy5.5-, APC, or unconjugated antibodies and conjugates were used in the experiments in appropriate combinations: rat anti-mouse F4/80 APC, clone BM8, dilution 1 : 100, rat anti-mouse CD11b PerCp-Cy5.5, clone M1/70, dilution 1 : 100 (both eBioscience, Frankfurt am Main, Germany), rat anti-mouse CD206 Alexa488, clone MR5D3, dilution 1 : 100 (BioLegend, Fell, Germany), anti-mouse iNOS FITC, clone 6/iNOS, dilution 1 : 50; anti-mouse Arg1 purified, clone 19/arginase-1, dilution 1 : 100, and rt anti-mouse IgG1, clone A85-1 (all BD Bioscience, Heidelberg, Germany). For intracellular staining, cells were fixed and permeabilized using the Fixation and Permeabilization Kit (Invitrogen, ThermoFisher Scientific, Waltham, USA) according to the manufacturer's instructions. Stained cells were analyzed on a FACS Canto II (BD Bioscience). Data analysis was performed using FlowJo 7.6.2 software (TreeStar, Ashland, OR, USA).

### 2.6. Phagocytosis Assay

Phagocytic activity was assessed using the Phagocytosis Assay Kit (IgG FITC) (Cayman Chemicals, Ann Arbor, USA) according to the instructions of the manufacturer. Briefly, activated BMDM were cultured in RPMI 1640/5% charcoal absorbed FCS at a concentration of 5 × 10^5^ cells/mL in a 24-well plate and exposed to S1P concentrations of 0–1000 nM (Sigma-Aldrich Chemie GmbH, Munich, Germany) for 12 h. Subsequently, 50 *μ*L of fluorescent rabbit IgG latex particles was added to each sample and incubation was continued for another 36 h. Subsequently, cells were harvested, washed, and analyzed by FACS using a FACS Canto II. All samples were run in triplicate. For statistical analysis median fluorescence values were used.

### 2.7. BMDM Migration In Vitro

Chemotaxis of M1- or M2-polarized BMDM was quantified using 8 *μ*m ThinCert cell culture inserts (Greiner Bio-One, Frickenhausen, Germany) in 24-well plates according to the manufacturer's recommendation. Cells that migrated to the bottom side of the membrane in 4 h at 37°C following various S1P gradients were stained with 8 *μ*M Calcein-AM fluorescent dye (Sigma-Aldrich Chemie GmbH, Munich, Germany). Fluorescence was measured by photometry at a wavelength of 485 nm/520 nm.

### 2.8. Cytokine Secretion Assay

To assess cytokine secretion polarized BMDM were cultured at a concentration of 5 × 10^5^ cells/mL in 500 *μ*L of RPMI 1640/5% charcoal absorbed FCS in the presence of various concentrations of S1P (0–100 *μ*M) for 24 h. Cell culture supernatants were then harvested and assayed for cytokine concentrations (IL-6, IL-12, TNF-*α*, MCP-1, and IL-10) using the BD Cytometric Bead Array Mouse Inflammation Kit (BD Bioscience, Heidelberg, Germany) according to the manufacturer's instructions. Data analysis was performed using the FCAP-Array 1.0.1 software (Soft Flow Inc., St. Louis Park, USA).

### 2.9. Statistical Analysis

SPSS 17.0 (SPSS Inc., Chicago, IL, USA) and GraphPad PRISM® 5 (GraphPad Software Inc.) were used for statistical analysis. Intergroup comparisons were performed using a one way ANOVA + Bonferroni's post hoc test. Intragroup comparisons were calculated using a one way ANOVA + Dunnett's test. Differences were considered significant at a *p* value of less than 0.05.

## 3. Results

### 3.1. Influence of Macrophage Polarization on S1PR Expression

**Figure 1:**
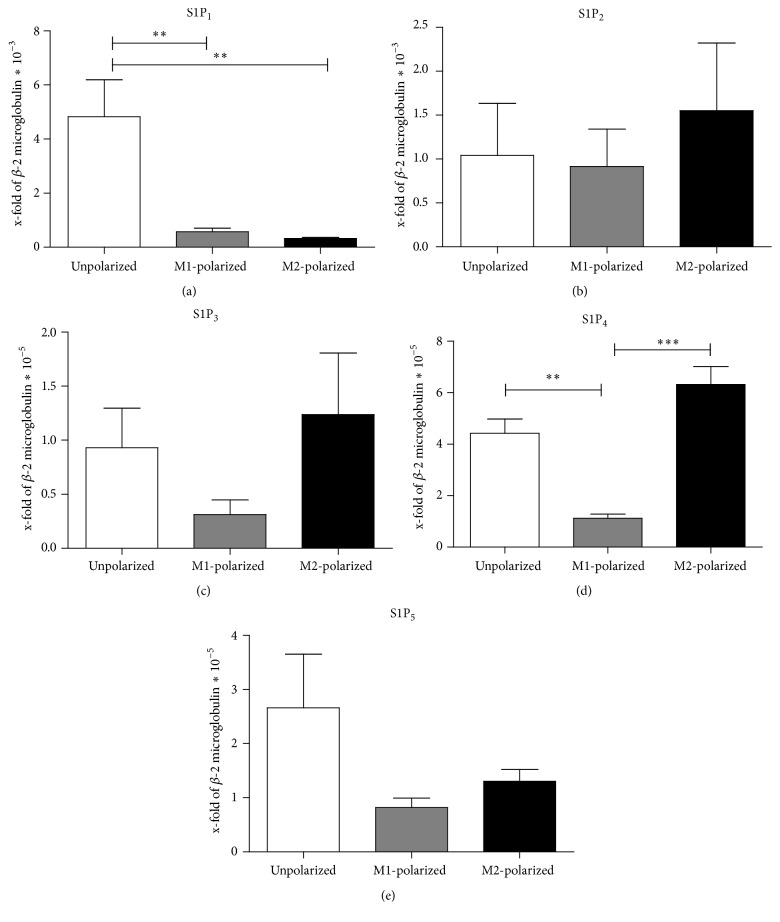
S1P-receptor expression in unpolarized, M1-polarized, and M2-polarized BMDM. Relative mRNA levels for S1P_1_ (a), S1P_2_ (b), S1P_3_ (c), S1P_4_ (d), and S1P_5_ (e) compared to *β*2-microgloblin expression were assessed using the ΔΔcT-method (*n* = 5 in 2 independent experiments). Data were reported as means ± SEM. ^*∗∗*^*p* < 0.01, ^*∗∗∗*^*p* < 0.001.

The biological cellular response to S1P depends on the expression profiles of 5 membrane bound S1P receptors (S1PR) in an individual cell. Since the characterization of the expression patterns of S1PRs on macrophages in the literature is incomplete and partially contradictory, S1PR expression in nonpolarized, M1-polarized, and M2-polarized BMDM was assessed by real-time RT-PCR. For this purpose, macrophages were differentiated from murine bone marrow and polarized towards the M1 or M2 phenotype by incubation with LPS in combination with IFN or IL-13 and IL-4, respectively. Expression of mRNAs of all 5 S1P-receptors was found in M1- and M2-polarized as well as in unpolarized macrophages (Figures 1(a)–1(e)). While the receptors S1P_2_, S1P_3_, and S1P_5_ showed similar expression levels in M1, M2, and unpolarized macrophages, S1P_1_ receptor expression was significantly reduced in M1- and M2-polarized macrophages compared to unpolarized macrophages (*p* = 0.0012). However, expression levels of S1P_1_ on M1- and M2-polarized macrophages were similar (*p* = 0.1508) (Figure 1(a)). In contrast, S1P_4_ expression was maintained during M2 polarization, while it was significantly reduced in M1-polarized macrophages (*p* < 0.0001) (Figure 1(d)).

### 3.2. S1P Favours Expression of the M1-Marker iNOS under M1-Polarizing Conditions

**Figure 2:**
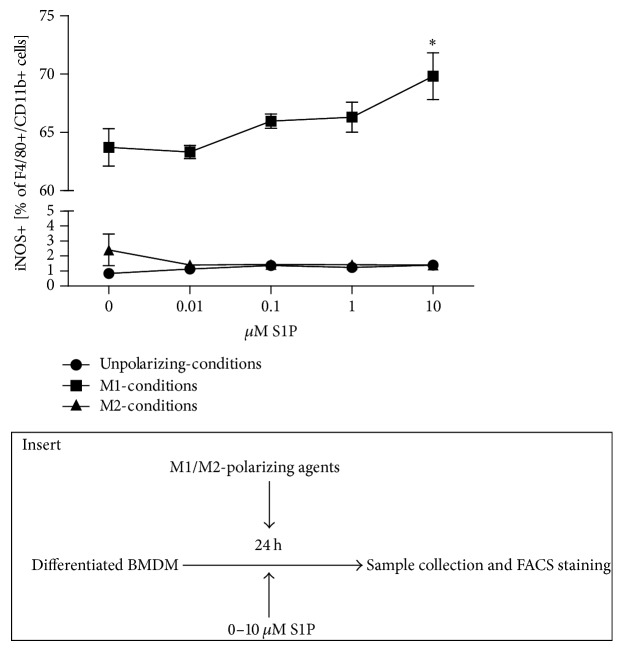
S1P influence on macrophage polarization. iNOS expression was assessed in BMDM cultured under M1- or M2-polarizing conditions in presence of various S1P concentrations (*n* = 5 in 2 independent experiments). Data were reported as means ± SEM. ^*∗*^*p* ≤ 0.05. Insert: scheme of the experimental design.

Since S1P receptors are present on differentiated but nonpolarized BMDM we wondered whether S1P may affect the efficacy of macrophage polarization under typical M1- and M2-polarizing conditions. After 24 hours of differentiation, BMDM were incubated under M1-polarizing conditions (1 *μ*g/mL LPS + 300 U/mL IFN*γ*) or M2-polarizing conditions (40 ng/mL IL-13 + 40 ng/mL IL-4) and increasing concentrations of S1P for additional 24 h. At the end of the incubation period, the percentage of macrophages expressing the M1-marker iNOS was significantly increased with rising concentrations of S1P (Figure 2) when cultured under M1-polarizing conditions (*p* = 0.019). As expected, the low percentage of iNOS expressing macrophages under M2-polarizing conditions remained unchanged (*p* = 0.521) (Figure 2). In contrast, the percentage of macrophages expressing the M2-marker arginase-1 remained constant with increasing concentrations of S1P under both M1- and M2-polarizing conditions (data not shown).

### 3.3. S1P Did Not Impact on the Expression of the M1 Surface Marker iNOS on Previously Polarized BMDM

**Figure 3:**
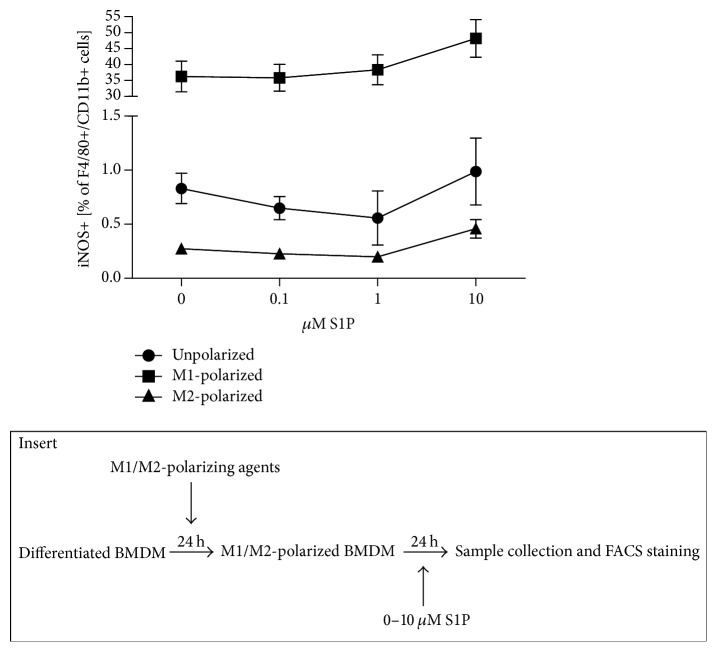
S1P influence on the expression of M1-markers in polarized macrophages. iNOS expression in presence of various S1P concentrations was assessed in previously polarized macrophages (*n* = 5 in 2 independent experiments). Data were reported as means ± SEM. Insert: scheme of the experimental design.

Following the observation that S1P favoured the expression of the M1 surface marker iNOS during the differentiation process under M1-polarizing conditions, we wondered whether S1P affects the phenotype of M1- and M2-polarized BMDM. After 24 hours of polarization with 1 *μ*g/mL LPS and 300 U/mL IFN*γ*, cultures of M1-polarized BMDM were exposed to increasing concentrations of S1P in the absence of further M1-polarizing cytokines. Percentages of iNOS expressing macrophages increased with higher S1P concentrations, but the tendency did not reach statistical significance (*p* = 0.2657) (Figure 3). M2-polarized BMDM (24-hour culture with 40 ng/mL IL-13 + 40 ng/mL IL-4) showed very low iNOS expression which remained unchanged with rising S1P levels in the absence of further M1- or M2-polarizing chemokines (Figure 3).

### 3.4. Chemotaxis of M1, M2, and Unpolarized Macrophages Is Differentially Affected by S1P

**Figure 4:**
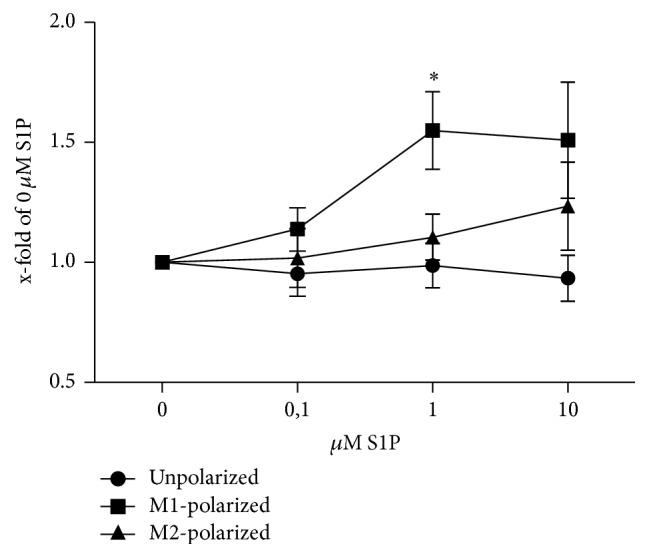
Chemotaxis to S1P of unpolarized, M1-polarized, and M2-polarized BMDM in vitro (*n* = 5 in 2 independent experiments). Data were reported as means ± SEM. ^*∗*^*p* ≤ 0.05.

Migration to sites of inflammation and tissue repair is a fundamental biological characteristic of macrophages. In order to assess the chemotactic potential of S1P on unpolarized and M1- and M2-polarized macrophages, we assessed their chemotactic response to rising S1P gradients in vitro. Unpolarized macrophages and M2-polarized macrophages showed no chemotactic response to various S1P gradients. However, M1-polarized macrophages showed a clear chemotactic response to rising S1P gradients which was concentration-dependent (*p* = 0.0233) (Figure 4). Maximal chemotactic response was reached at a concentration of 1 *μ*M S1P and was not further enhanced with higher S1P concentrations.

### 3.5. S1P Signalling Did Not Influence the Phagocytic Activity of M1- and M2-Polarized Macrophages

**Figure 5:**
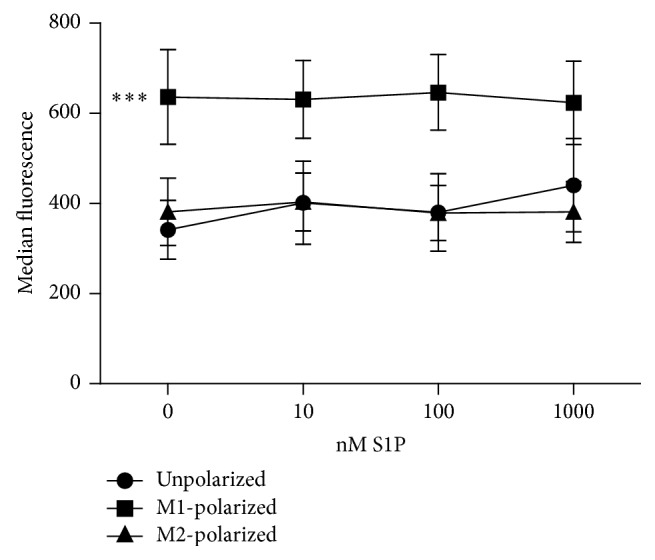
S1P influence on phagocytic capacity of BMDM. Phagocytic activity of unpolarized, M1-polarized, and M2-polarized BMDM was tested in presence of growing S1P-concentrations in vitro (*n* = 5 in 1 independent experiment). Data were reported as means ± SEM. ^*∗∗∗*^*p* < 0.001.

Phagocytic activity is a major biological characteristic of macrophages. As expected, M1-polarized macrophages exhibited a higher phagocytic capacity for rabbit IgG coated latex particles than M2-polarized or nonpolarized BMDM. In all three experimental groups, phagocytic activity remained constant over a wide range of S1P concentrations in the culture medium (0–1000 nM) (Figure 5). Thus, S1P signalling did not influence the phagocytic activity in macrophages.

### 3.6. S1P Induced Increased Production of Proinflammatory Cytokines in M2-Polarized and Unpolarized BMDM

**Figure 6:**
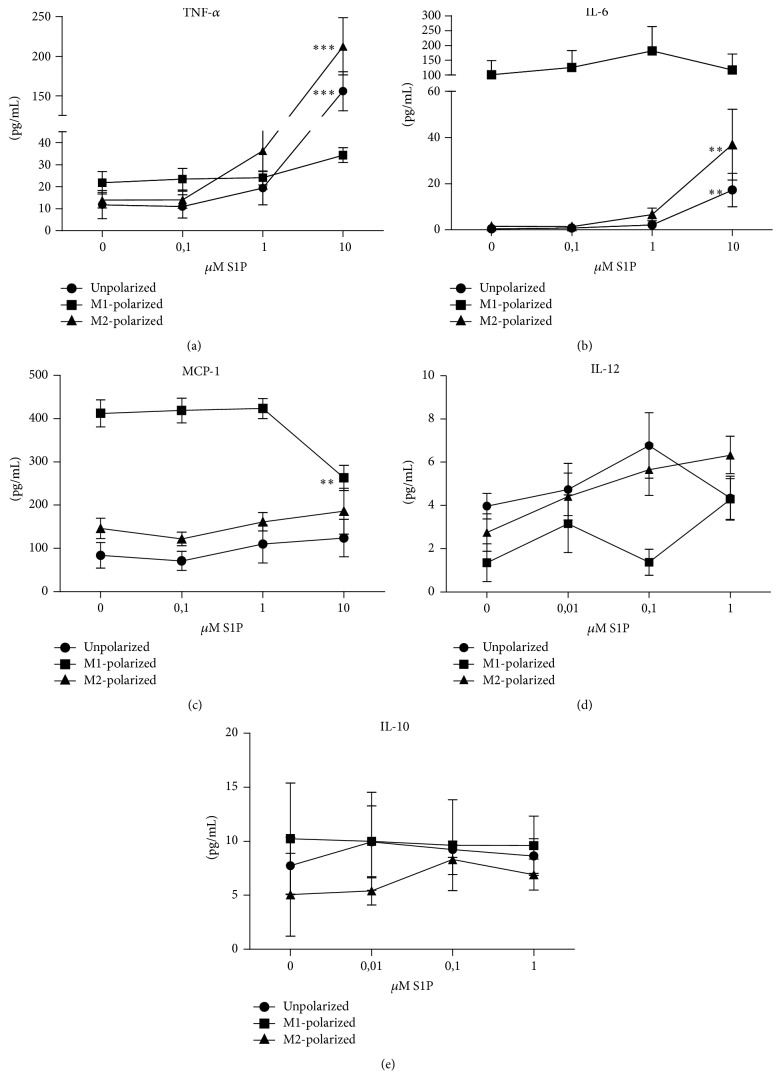
S1P influence on BMDM cytokine production. Cytokine production in unpolarized, M1-polarized, and M2-polarized BMDM in presence of various S1P-concentrations in vitro was assessed by CBA: TNF-*α* (a), IL-6 (b), MCP-1 (c), IL-12 (d), and IL-10 (e) (*n* = 5 in 4 independent experiments). Data are reported as means ± SEM. ^*∗∗*^*p* < 0.01, ^*∗∗∗*^*p* < 0.001.

To investigate whether S1P signalling influences inflammatory processes by modulating cytokine production of macrophages, exogenous S1P was added to cultures of previously in vitro polarized M1, M2 or unpolarized macrophages. Production of proinflammatory cytokines (IL-6, IL-12, TNF-*α*, and MCP-1) and anti-inflammatory cytokines (IL-10) was measured in the culture supernatant. In M1-polarized macrophage cultures, an increase of S1P did not influence secretion of proinflammatory cytokines (Figures 6(a)–6(d)). Also, production of IL-10 was not altered by S1P in M1-polarized macrophage cultures (Figure 6(e)). Interestingly, both previously M2-polarized macrophages and unpolarized BMDM produced increasing levels of the proinflammatory cytokines TNF-*α* (*p* < 0.0001 and *p* < 0.001, resp.) and IL-6 (*p* = 0.0066 and *p* = 0.006, resp.) when exposed to increasing concentrations of S1P (Figures 6(a) and 6(b)).

## 4. Discussion

The effect of S1P on cellular functions of immune cells is the net effect of the relative expression of the S1P receptor subclasses on individual cells [28]. It has been shown that the expression pattern of S1P receptors may change during differentiation and activation of an individual cell. Thus, S1P_1_ and S1P_3_ were substantially upregulated in in vitro matured dendritic cells (DC) compared to immature DC, probably accounting for the differences in the migrational response to S1P gradients observed between these two maturation states [29]. In mast cells, activation through binding of the Fc*ε*RI receptor resulted in upregulation of S1P_2_ expression, leading to reduced migrational responses to S1P and to local accumulation of activated mast cells [30].

S1P levels in acutely inflamed tissues were significantly increased [31] and S1P has been shown to be released by apoptotic cells [32]. In patients with systemic inflammation, S1P levels in plasma were shown to be of prognostic value [33]. Thus, S1P is a promising candidate as a regulator of local and systemic inflammatory processes.

Murine bone marrow derived macrophages (BMDM) are a frequently used and well defined source of macrophages in experimental research [5]. In the present paper we could show that all S1P receptor subclasses were expressed on BMDM. Characterization of S1P expression patterns of macrophages has shown differing results, most likely due to the heterogeneity of macrophage populations used in different studies [17–21, 23, 34]. While expression of S1P_1_ and S1P_2_ could be consistently shown in murine and human macrophage populations, expression of the remaining three S1P receptor subclasses was reported more inconsistently. However, results of comparative analysis of S1P receptor expression profiles in different macrophage polarization states are scarce. Interestingly, comparison of S1P receptor expression patterns showed significant differences between nonpolarized, M1-polarized, and M2-polarized BMDM. These differences were found in the expression of the S1P receptors S1P_1_ and S1P_4_. S1P_1_ was expressed on BMDM before polarization, and S1P_1_ levels were significantly downregulated in M1- as well as in M2-polarized BMDM. Interestingly, Weichand et al. described modification of S1P_1_ expression in human primary macrophages after exposure to classical M2-polarizing agents (IL-4 and apoptotic cell supernatant) [23]. This process was time dependent and reached its maximum after 6 hours. We attribute these divergent observations to the difference in sources of macrophages used in the experimental settings, that is, human primary cells versus murine BMDM. S1P_4_ expression was significantly downregulated in M1-polarized BMDM compared to unpolarized BMDM. Interestingly, in M2-polarized BMDM S1P_4_ expression was maintained on similar levels compared to unpolarized BMDM. Therefore, this receptor is a promising candidate to account for functional differences in the response of M1- and M2-polarized macrophages when exposed to S1P.

S1P signalling was shown to shift macrophage polarization towards M2 anti-inflammatory phenotype in previously unpolarized macrophages and under otherwise nonpolarizing conditions [10, 19, 35]. The temporal sequence of activating and polarizing stimuli seems to be of importance in macrophage polarization as shown for IL-4 and LPS [36]. We thus wondered whether the presence of S1P might modify the efficiency of macrophage polarization in presence of typical M1- as well as M2-polarizing cytokines. Interestingly, the expression of iNOS as a typical M1 marker was further enhanced by coincubation with increasing S1P levels under M1-polarizing conditions, while expression of the M2-marker arginase was not further increased in presence of S1P under M2-polarizing conditions. The induction of an anti-inflammatory phenotype in macrophages was reported to be mediated by S1P_1_ [10, 19]. However, we could not find an effect of S1P on macrophage polarization under M1- and M2-polarizing conditions. This may be due to the downregulation of S1P_1_ expression in polarized macrophages.

Both M1 and M2 polarization do not represent an end-stage of macrophage differentiation. Instead, the two states display high plasticity and may be interconverted by appropriate molecular signals [9]. This plasticity allows macrophages to change from one functional phenotype to another when environmental conditions change, that is, during different phases of disease processes or wound healing. We therefore assessed whether S1P may have an impact on the phenotype of macrophages previously polarized by exposure to M1- or M2-polarizing cytokines. Interestingly, neither M1-polarized nor M2-polarized macrophages showed changes in expression of the marker-proteins arginase and iNOS upon exposure to increasing S1P concentrations after previous M1 and M2 polarization.

Although iNOS expression as a marker for M1 polarization was not affected by exposure to increasing S1P levels during culture under M2-polarizing conditions, we observed a positive effect of S1P on the secretion of proinflammatory cytokines (IL-6 and TNF-*α*) in BMDM cultured with IL-4/IL-13 favouring M2 polarization as well as in unpolarized macrophages. Apoptotic tumor cell supernatant containing elevated levels of S1P was reported to selectively alter the production of TNF-*α* and IL-8, while IL-10 production was not altered [35]. The S1P receptor responsible for the mediation of this effect has not been identified yet. Interestingly, the only S1P receptor with significantly different expression levels in M1- and M2-polarized BMDM is S1P_4_ showing significantly increased expression levels in M2-polarized BMDM. It has been earlier shown that S1P_4_ deficient dendritic cells show reduced IL-6 production in vitro and that S1P_4_ deficient mice had lower IL-6 levels in a colitis model in vivo [15]. Although various S1P receptors may influence cytokine secretion by macrophages, our observations indicate that the differences of cytokine secretion in response to increasing S1P concentrations observed in M1 macrophages on the one hand and M2-polarized macrophages/unpolarized BMDM on the other hand are mediated by the S1P receptor differentially expressed in these cell populations. This receptor is the S1P_4_.

Neither unpolarized nor M1- and M2-polarized macrophages showed a significant change of phagocytic activity with increasing extracellular concentrations of S1P. S1P receptor-mediated signalling has been described to influence phagocytosis of various pathogens though, albeit with opposing effects. While McQuiston et al. report that S1P_2_-mediated S1P-signalling facilitates phagocytosis of antibody-opsonized* Cryptococcus neoformans* by alveolar macrophages [37], Hou et al. elegantly showed that S1P_2_ signalling impaired phagocytosis of* Escherichia coli* in vitro as well as in an animal model [38]. The observed differences are most likely due to different mechanisms/receptors implicated in the receptor-mediated phagocytosis of fungi, gram-negative bacteria, and antibody-opsonized foreign bodies [39]. In our model, S1P_2_ expression levels were similar in unpolarized and M1- and M2-polarized macrophages, making S1P-mediated differences in the phagocytic capacity less likely.

S1P production is significantly increased in cells undergoing apoptosis [40]. S1P has further been shown to be a potent chemoattractant for monocytic cells lines as well as human primary monocytes and macrophages [40]. Based on these observations, S1P is thought to be a lipid attraction signal released during apoptosis to guide macrophages to tissues where increased cell death by apoptosis occurs in order to efficiently eliminate cells undergoing apoptotic death. Here we could show that M1-polarized macrophages were more sensitive to the chemoattractant activity of S1P than M2-polarized or unpolarized macrophages. Reports from several groups have implicated S1P_1_ and S1P_2_ in macrophage migration. While S1P_1_ was shown to increase macrophage migration [41], S1P_2_ was reported to have an inhibitory action [18]. However, M1- and M2-polarized macrophages differed primarily in S1P_4_ expression levels. We could previously show that S1P_4_ deficient lymphocytes showed an increased migration to S1P gradients in vitro and in vivo. This observation suggests an inhibition exerted by S1P_4_-mediated S1P signalling on chemotaxis mediated via S1P_1_ [15]. We now observed a similar constellation in polarized macrophages. This observation indicates that S1P_4_ might inhibit S1P_1_-mediated chemotaxis in response to S1P. Thus, reduced S1P_4_ expression levels in M1-polarized macrophages compared to M2-polarized macrophages in presence of similar expression levels of S1P_1_ result in increased migrational response of M1-polarized macrophages to S1P. This increased migration of M1-polarized macrophages to S1P based on an increased S1P_1_/S1P_4_ ratio may be implicated in the resolution of the inflammatory infiltrate. In accordance with this hypothesis, S1P_1_-deficient macrophages showed reduced emigration from the site of inflammation in a model of peritoneal inflammation [23].

## 5. Conclusion

In summary, we could show that M1- and M2-polarized macrophages expressed different S1P receptor profiles. Furthermore, they showed different responses to S1P gradients concerning chemotactic migration and cytokine secretion. The involvement of S1P as a regulatory molecule in a myriad of physiological and pathological conditions stimulated the development of a large number of pharmacological compounds affecting the S1P signalling axis [42]. The expression of differential S1P receptor profiles may provide a possibility to more selectively influence M1- or M2-polarized macrophages, for example, tumor associated macrophages. Further in vivo experiments involving S1P_4_^−/−^ animals are required to corroborate the physiological relevance of the differential S1P profiles in macrophage populations observed in vitro.

## Supplementary Material

In order to demonstrate successful macrophage polarization, culture samples were stained with anti-CD206 to assess M2-polarization and anti-iNOS to assess M1 polarization.
